# Re-Shuffling of Species with Climate Disruption: A No-Analog Future for California Birds?

**DOI:** 10.1371/journal.pone.0006825

**Published:** 2009-09-02

**Authors:** Diana Stralberg, Dennis Jongsomjit, Christine A. Howell, Mark A. Snyder, John D. Alexander, John A. Wiens, Terry L. Root

**Affiliations:** 1 PRBO Conservation Science, Petaluma, California, United States of America; 2 Climate Change and Impacts Laboratory, Department of Earth and Planetary Sciences, University of California Santa Cruz, Santa Cruz, California, United States of America; 3 Klamath Bird Observatory, Ashland, Oregon, United States of America; 4 Woods Institute for the Environment, Yang & Yamazaki Environment & Energy Building, Stanford University, Stanford, California, United States of America; American Museum of Natural History, United States of America

## Abstract

By facilitating independent shifts in species' distributions, climate disruption may result in the rapid development of novel species assemblages that challenge the capacity of species to co-exist and adapt. We used a multivariate approach borrowed from paleoecology to quantify the potential change in California terrestrial breeding bird communities based on current and future species-distribution models for 60 focal species. Projections of future no-analog communities based on two climate models and two species-distribution-model algorithms indicate that by 2070 over half of California could be occupied by novel assemblages of bird species, implying the potential for dramatic community reshuffling and altered patterns of species interactions. The expected percentage of no-analog bird communities was dependent on the community scale examined, but consistent geographic patterns indicated several locations that are particularly likely to host novel bird communities in the future. These no-analog areas did not always coincide with areas of greatest projected species turnover. Efforts to conserve and manage biodiversity could be substantially improved by considering not just future changes in the distribution of individual species, but including the potential for unprecedented changes in community composition and unanticipated consequences of novel species assemblages.

## Introduction

With rapid climate disruption, many species may adapt by shifting their ranges independently of other species [1], [2]. This differential movement is evidenced by the existence of major North American and European plant and animal assemblages with no modern analogs as recently as 10,000 BP [3]–[5]. Even within the last century, significant changes in community composition have been attributed to climate change [6], [7]. Such changes will likely become more extreme because warming is predicted to escalate for at least the next 40 yr [8], potentially resulting in novel combinations of species. Consequently, current community dynamics such as predator-prey or competitive interactions may become affected as species assemblages are reshuffled in new ways [9]. New species interactions that develop within these no-analog assemblages may result in the decline or extirpation of species as they adjust or adapt to changing climates, especially when the climate is changing at a rapid rate. Realizing the need to understand the possibility of unexpected responses resulting from changes in species co-occurrence led us to identify no-analog future communities by developing a systematic quantification of potential climate-induced community changes for California's terrestrial breeding birds.

Despite their limitations [10]–[12], species-distribution models (SDMs) allow us to project the effects of future climate change on the occurrence patterns of species, ecosystems, and biomes. Many studies have projected future changes in species' distributions and patterns of diversity, with an emphasis on range contractions and the potential for species extinctions [13]–[15]. Most such studies have identified more range contractions than expansions. To synthesize multi-species impacts of climate disruption, however, one must take the next step of considering changes in patterns of species co-occurrence. Several studies have done this by quantifying expected rates of species turnover as an indication of change in community composition [10], [16]–[18]. Although high rates of projected species turnover have been identified for many geographic areas, this does not directly consider the degree to which novel or “no-analog” communities may be anticipated. Entirely unique combinations of species and the new interactions that occur among those species may lead to even greater rates of local extirpation if species cannot adapt quickly enough [19].

We focused on California because it is a large, floristically and topographically diverse state that global climate models (GCMs) have shown to be particularly vulnerable to the effects of a changing climate [20], [21]. Climate models generally concur in projections of significant warming for California over the next century, with small changes in precipitation but potentially large declines in snow accumulation [22], [23]. The diverse climate and topography of the state can be represented in regional climate models (RCMs), which use fine-scale horizontal resolutions and specific physics and parameterizations to dynamically downscale GCM predictions [23]. Although statistically downscaled GCMs also yield improved estimates at a relatively fine scale [24], we chose to use computationally-intensive RCMs because they can simulate nonlinear climate processes that are likely to change in the future.

We used a representative subset of terrestrial breeding birds to evaluate the potential for no-analog assemblages as a result of projected climate disruption. We chose birds for this analysis due to their high trophic position, relatively high visibility and detectability during the breeding season, and high mobility, which we assume allow them to track environmental change rapidly [25], [26]. We used high-quality, breeding-season datasets from multiple sources to develop intermediate-scale (800-m pixel resolution) spatial models to predict current and future probabilities of occurrence for each of 60 focal species selected to represent avian communities of five major habitat types: oak woodland, coniferous forest, chaparral/scrub, grassland, and riparian [27].

At the continental scale, avian distribution models are typically based on temperature- and precipitation-based bioclimatic variables [28], [29]. These factors may limit bird distributions directly, via physiology, but they also help determine habitat availability for birds, via vegetation patterns. At a regional level, however, the inclusion of vegetation/landcover in SDMs can be used to create more refined projections [30], [31]. This is especially true for regions of high topographic diversity, where SDMs must have sufficient resolution to predict how species and communities will respond to climate change in heterogeneous landscapes. Thus, to improve the capacity of our models to incorporate changes relevant to birds, we included vegetation distribution, modeled from climate, soil, and topographic variables for the future period. Based on the frequent finding that birds respond more to the structure and form of vegetation than to floristic composition [32], we used general vegetation types (aggregations of classified vegetation types at the state level) rather than plant species to represent bird habitats. By focusing on life form and habitat structure rather than plant species composition, we avoided making the unrealistic assumptions that individual plant species would be able to disperse to more climatically suitable areas in a short time-frame and that future vegetation communities would maintain their current assemblages of plant species. Although our SDM approach to vegetation modeling may omit some important mechanisms for vegetation change, such as direct physiological effects of CO_2_ [33] and changes in vegetation disturbance [34], we found existing mechanistic vegetation model outputs [20] too spatially coarse for our purposes.

We compared assemblages of bird species under current climatic conditions with predicted future assemblages based on RCM projections with inputs from two different GCMs (GFDL and CCSM, see Materials and Methods) under a medium-high emissions scenario [IPCC SRES A2 [8]] through 2070. Two SDM algorithms (GAM and Maxent, see Materials and Methods) were used for comparison purposes. We applied the modern analog method [35] of paleoecology, which has been used to compare pollen or fossil samples with modern assemblages [3], [36] and to identify future no-analog climates [37]. We quantified avian community dissimilarity for all possible combinations of current and future locations throughout California, based on predicted probabilities of individual species occurrences, to identify possible no-analog communities of the future. To accomplish this, we compared results across a range of dissimilarity thresholds based on different levels of community aggregation (i.e., “community scale”; 20–100 groups, see Materials and Methods). We also compared the geographic patterns of no-analog communities to patterns in local community change over time.

## Results

### Individual species predictions

Our models had very good predictive abilities for the current period (Table 1). On average, Maxent models predicted higher probabilities of current and future occurrence than did the GAMs. The mean change in species' mean state-wide probability of occurrence was also greater for Maxent-based predictions than for GAM-based predictions, as was the number of species whose distributions were predicted to decrease. The mean predicted absolute change in species' distributions was greater for the GFDL-based climate projections (41–43%) than for the CCSM-based projections (29–32%), although more species were predicted to decrease based on CCSM compared to GFDL. All SDM-climate-model combinations predicted more species decreasing than increasing.

**Table 1:** Summary of current and future species distribution model predictions.

SDM algorithm	Mean AUC^1^	Mean current *P* 2	Climate model	Mean future *P* 2	Mean change (*P*/%)^3^	# species increasing/decreasing
GAM	0.904	0.123	GFDL CM 2.1	0.124	0.0410/42.9%	22/38
			NCAR CCSM 3.0	0.113	0.0303/31.8%	17/43
Maxent	0.895	0.177	GFDL CM 2.1	0.146	0.0583/41.3%	13/47
			NCAR CCSM 3.0	0.149	0.0403/28.8%	9/51

1 Area under the curve of the receiver operating characteristic plot.

2 Predicted probabilities of occurrence, averaged over 637,290 800-m grid cells and 60 species (Table S1). Mean change across species is based on absolute values of individual species' mean change across all pixels.

3 (future *P* – current *P*)/current *P.*

### No-analog communities

**Figure 1:**
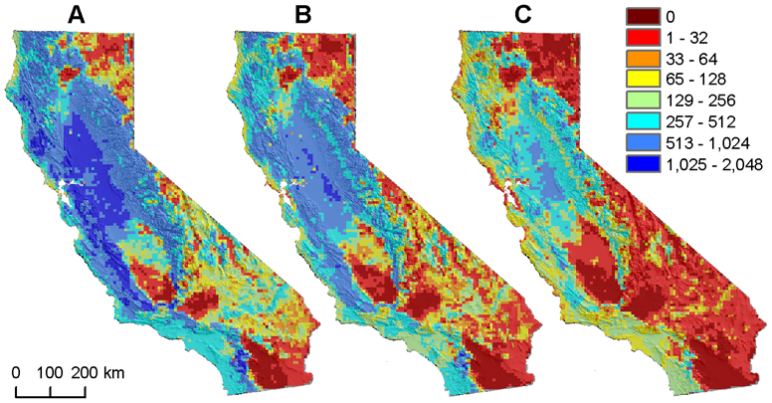
Number of modern analogs for predicted future bird communities across scales. “Analog” communities are those for which Bray-Curtis dissimilarity was less than an ROC-determined optimal threshold, based on the level of community aggregation: A, 20 groups. B, 60 groups. C, 100 groups. Here, predictions of future bird communities are based on GFDL CM2.1, Scenario A2, 2038–2070, generalized additive models. Patterns across scales were similar for the other climate models and distribution-model algorithms.

**Figure 2:**
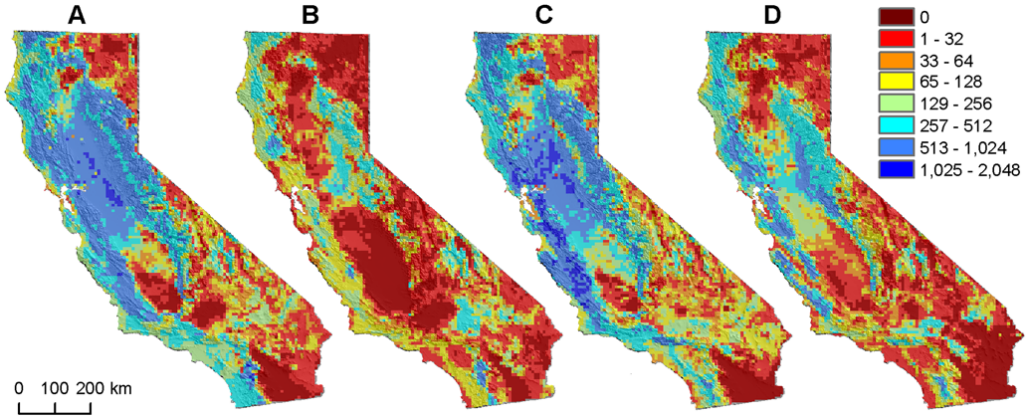
Number of modern analogs for predicted future bird communities across climate models and distribution-model algorithms. “Analog” communities are those for which Bray-Curtis dissimilarity was less than an ROC-determined optimal threshold, based on a 60-group level of community aggregation. Predictions of future bird communities are based on: A, GFDL CM2.1, Scenario A2, 2038–2070, generalized additive models. B, GFDL CM2.1, Scenario A2, 2038–2070, maximum entropy models. C, NCAR CCSM3.0, Scenario A2, 2038–2069, generalized additive models. D, NCAR CCSM3.0, Scenario A2, 2038–2069, maximum entropy models.

Looking at future predicted bird communities across all combinations of climate models, SDM algorithms, and community scales, estimates of no-analog communities varied considerably, ranging from 10% to 57% of California's land area (Table 2). For any given combination of climate model and SDM algorithm (e.g., GFDL and GAM, Fig. 1), the frequency of no-analog grid cells increased with the number of grouping levels considered. At the broadest community scale evaluated (20 groups), 10% to 25% of grid cells had effectively no modern analogs (<0.5% of total area, see Materials and Methods), depending on the climate model and SDM algorithm. At the finest scale of community composition evaluated (100 groups), the estimate ranged from 37% to57%. For our intermediate grouping level (60 groups), 18% to 50% of the state had effectively no modern analogs. These areas occurred primarily in the non-coastal portions of the state (Fig. 2).

**Table 2:** Percent of future predicted California bird communities with no modern analogs^1^.

	Number of groups			
Climate model/Distribution model algorithm	100	60	20	5
Current/Maxent (baseline)	7.11%	4.69%	0.941%	0%
Current/GAM (baseline)	8.33%	2.27%	0.863%	0.0628%
GFDL CM2.1/Maxent	56.6%	50.0%	25.1%	1.49%
GFDL CM2.1/GAM	41.2%	23.1%	13.6%	3.26%
NCAR CCSM3.0/Maxent	46.7%	40.3%	20.7%	2.81%
NCAR CCSM3.0/GAM	37.2%	18.3%	9.57%	3.48%

1 Values are expressed as the percent of total land area, standardized with respect to grid cell resolution, in that any grid cell with <32 modern analogs (<0.5% of the total area) was considered to have effectively no modern analog.

The magnitude of community change varied more by SDM than by climate model, with Maxent-based estimates of no-analog bird communities being more dramatic than those based on GAMs. Geographic patterns exhibited similar levels of variation across climate models and SDMs, with GFDL-based projections generally more dramatic and spatially clustered. Areas of agreement across climate models and SDMs in the distribution of no-analog communities occurred primarily in the southern deserts, in the southern portion of California's central valley, and in the northeastern portion of the state (Klamath Mountains and Modoc Plateau). Areas projected to have the greatest number of modern-analog communities tended to be located in central California around the San Francisco Bay/Delta region; intermediate values were found throughout the coastal and Sierra Nevada mountain ranges.

### Species Turnover

**Figure 3:**
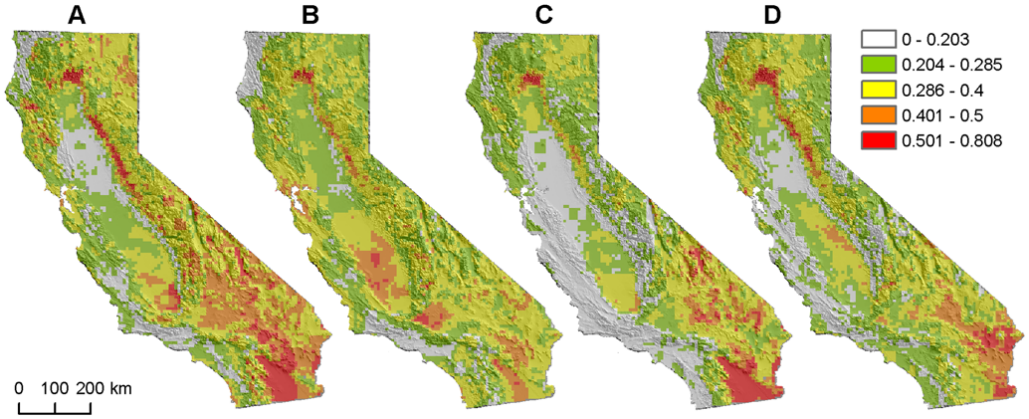
Predicted species turnover across climate models and distribution-model algorithms. Species turnover was calculated as the Bray-Curtis (BC) dissimilarity between predicted current and future bird communities. Lower category breaks (0.204, 0.285) represent optimal dissimilarity threshold used to identify non-analog communities, based on a 60-group level of community aggregation (GAMs and Maxent models, respectively). Predictions of future bird communities are based on: A, GFDL CM2.1, Scenario A2, 2038–2070, generalized additive models. B, GFDL CM2.1, Scenario A2, 2038–2070, maximum entropy models. C, NCAR CCSM3.0, Scenario A2, 2038–2069, generalized additive models. D, NCAR CCSM3.0, Scenario A2, 2038–2069, maximum entropy models.

All of the areas that were predicted to have no-analog communities under future climate change scenarios were, by definition, also identified as areas of high species turnover, based on pixel-level community dissimilarity (Fig. 3). The reverse was not true, however, as many of the areas of greatest predicted change in community composition—primarily located in mountain regions such as the northern Sierra Nevada foothills—had future predicted bird communities with many modern analogs.

## Discussion

Our analysis suggests that, by 2070, individualistic shifts in species' distributions may lead to dramatic changes in the composition of California's avian communities, such that as much as 57% of the state (based on the scales of communities that we examined) may be occupied by novel species assemblages. An even greater area would be considered no-analog if finer community delineations were considered. Furthermore, because we used a representative subset of California terrestrial breeding birds, it is likely that we underestimated the frequency of no-analog bird communities that would be apparent if greater ecological complexity were considered. Thus, although net changes in the distributions of common species may be relatively small due to the combination of local decreases and increases, the cumulative effect on community composition is likely to be great due to variation in individual species' responses to climate disruption and resulting differences in geographic shifts.

Our conclusions differ from those of a similar analysis of European birds [38]. This is not surprising given the different scales of analysis (fewer than 25 species groupings were evaluated for all of Europe). Our results do not necessarily suggest that entirely novel California biomes (as indicated by birds), on the order of no-analog biomes identified for the late-Pleistocene [3], [5], would be anticipated by the year 2070. Rather, our model-based findings highlight the potential for new patterns of community assembly across a range of local and regional scales over the 60-year timeframe of this study. Although our study focused on birds, the situation may be similar for other taxa [39].

Because our numeric findings are scale-dependent, in terms of how a community is defined, one should not focus on specific percentages of predicted no-analog areas, but instead consider the geographic patterns over a range of community scales. Based on the most refined delineation of communities, our analysis revealed several no-analog “hotspots,” primarily in arid inland portions of the state. These patterns may reflect the greater climatic variability of inland areas with continental climates and little or no moderating maritime influence, which are also likely to be more influenced by climate disruption [21]. In addition, regions of high geologic diversity such as the Klamath Mountains in northern California, which represent the convergence of three mountain ranges [40], may also have high bird community heterogeneity and thus greater potential for the re-shuffling of species.

Although the “no-analog” future communities that we identified may indeed have analogs outside the state (perhaps especially in Baja California, Mexico), they still represent novel communities for California, and as such may pose significant management challenges for agencies or groups not accustomed to looking beyond state borders. On the other hand, we did not constrain our quantification of modern analogs within the state by distance, so a current community could be considered a modern analog of a future predicted community even if it were in a distant part of the state. Given California's high variability, however, these more distant “analogs” are more likely to differ in other respects not captured by our bird models. Thus, these sources of over- and under-estimation of modern analogs may partly balance each other out, even if geographic patterns are biased toward the southern parts of the state.

In contrast with species-turnover “hotspots” identified from the same set of model predictions, the emergence of novel communities was not generally predicted for mountainous regions of the state (notably the Sierra Nevada range), where communities might be expected to shift upslope in unison, maintaining the overall integrity of current species assemblages. Recent [7] and paleoecological [41] studies provide some evidence to the contrary, however. No-analog bird communities could arise in these areas for a number of reasons not captured by our distribution modeling approach, such as differential rates of upslope migration for different bird species, as well as for the individual plant or invertebrate species that they may rely upon for nesting or for food.

Our analysis assumes that species interactions do not constrain current or future species distributions. This is one of the chief limitations of an empirical SDM approach, which necessarily models the realized, rather than fundamental niche of a species [12]. The inclusion of vegetation in our bird models, however, may indirectly capture some of the factors that determine a species' realized niche [10]. Although our models could potentially have included existing species interactions (i.e., co-occurrence with other bird species [42]), we cannot predict interactions among previously unknown combinations of species. The novel communities that result from distributional shifts may persist as species adapt or coexist, or they may undergo further change as species are excluded through competition, predation, or other biotic interactions. Regardless of the outcome, these no-analog communities will be characterized by high levels of ecological change.

Taking a Gleasonian view of ecological communities [1], the high frequency of no-analog bird communities that may occur over the next century can be said to reflect the individualized nature of climate-change impacts on different species and the transient nature of current ecological communities as we know them [43], [44]. Previous research has recognized that climate change is likely to accelerate the reshuffling of current communities [9], and the effect has been demonstrated through modeling for individual sets of interacting species [45]. Here we have provided a systematic quantification of potential climate-induced community change for a large and diverse taxonomic group, using best-available datasets and species-distribution modeling techniques applied to focal species.

The likely emergence of novel, no-analog communities over the coming decades presents enormous conservation and management challenges. These challenges will be exacerbated in the high proportion of landscapes that are dominated by intensive human management [46], [47], where it will be more difficult for species to move to new climatically suitable areas. As new combinations of species interact, some species will face new competition and/or predation pressures while others may be released from previous biotic interactions. Managers and conservationists will be faced with difficult choices about how, where, and on which species to prioritize their efforts and investments. Traditional management approaches that focus on maintaining the status quo will not likely be successful; novel approaches will be needed to manage novel communities [48]. Adaptive management will become even more important as conservation targets shift and new ones emerge in unanticipated ways. Successful adaptive management will depend on rapid transfer of information from the scientific community to resource managers so that decisions can be made quickly. Scientists in a no-analog future will need to be more actively involved in planning and decision-making processes that affect biodiversity.

## Materials and Methods

### Avian Occurrence Data

Geo-referenced point-count survey [49] data were obtained from three sources: 1. PRBO Conservation Science (PRBO) and partners for 1993–2007 (http://www.prbo.org/cadc/); 2. USDA Forest Service Pacific Southwest Research Station Redwood Sciences Laboratory (RSL) and Klamath Bird Observatory (KBO) for 1992–2006; and 3. the North American Breeding Bird Survey (BBS) for 1997–2006 [50] (Fig. S1). Only BBS points with available GPS coordinates were used. The northern and central portions of the state were more comprehensively sampled than the southern part of the state. Several filters were applied to PRBO, RSL, and KBO point-count data to remove non-breeding records. Migratory species (Table S1) were only included only if the species was encountered on more than one survey within a season for a given survey route. An exception was made for the desert areas of southern California, which were surveyed earlier in the season, resulting in multiple detections of non-breeding migrants. For these points, we excluded all species known not to breed in the desert, regardless of encounter rate. All records were subsequently filtered to include only data from April through July. BBS surveys, which are conducted at the height of the breeding season (June), were assumed to represent breeding individuals.

We excluded all listed and special concern [51] species in order to focus on the relatively common species that are more likely to be climate- or vegetation-limited, rather than constrained by demography (e.g., low reproductive success or survival). This decision also enabled us to focus on species with comprehensive data.

### Climate Data

Current climate data were based on 30-year (1971–2000) monthly climate normals interpolated at an 800-m grid resolution by the PRISM Group [52]. We used monthly means for total precipitation and minimum and maximum temperature to derive a standard set of biologically meaningful climate variables [53].

Future climate scenarios were based on projections from a regional climate model, RegCM3 [54] at a 30-km resolution, with emissions trajectories taken from the Intergovernmental Panel on Climate Change (IPCC) SRES A2 scenario [8] and boundary conditions based on output from two GCMs:

1. CCSM: National Center for Atmospheric Research (NCAR) Community Climate System Model (CCSM3.0), an atmosphere-ocean global climate model (AOGCM) run from 1870–2099. The RCM time periods run were 1968–1999 (observed CO_2_) and 2038–2069 (478–610 ppm CO_2_).
2. GFDL: Geophysical Fluid Dynamics Laboratory (GFDL) GCM CM2.1, an AOGCM run from 1860–2099. The RCM time periods run were 1968–2000 (observed CO_2_) and 2038–2070 (478–615 ppm CO_2_).

RCM monthly temperature and precipitation outputs were averaged across years to obtain one set of monthly values for the current and future time windows. The delta values (difference for temperature, ratio for precipitation) between the current and future RCM values were applied to the PRISM climate grids to produce future monthly temperature and precipitation grids at an 800-m resolution.

### Vegetation Classification Model

We used current vegetation mapped by the California Gap Analysis Project [55] to model future vegetation based on observed relationships between vegetation, soil, climate, and topography. Vegetation types based on the California Wildlife Habitat Relationships System [56] were aggregated into 12 general classes to improve model classification accuracy (Table S2). We excluded developed and agricultural vegetation categories from our model, as well as aquatic, wetland, riparian, and non-vegetated categories that were thought to be driven more by proximity to water sources or were not directly climate-associated. Several uncommon vegetation types were also excluded due to sample-size limitations.

From a 10-km grid of points across the state, we removed those that fell in an excluded vegetation type and used the resulting sample (n = 9,752) to develop vegetation classification models using the Random Forest algorithm [57]. We used the ‘randomForest’ package for R [58], building 500 classification trees with three randomly-sampled candidate variables evaluated at each split.

As inputs to the vegetation models we used eight derived bioclimatic variables, three soil variables, and two topographic variables (Table S3). The resulting set of models was used to develop general vegetation predictions for the future time periods based on the CCSM and GFDL climate models (Fig. S2). Soil and topographic variables were assumed to remain the same in the future period. For consistency with the current vegetation layer, predicted future vegetation was augmented with the current urban and agricultural landcover types, which were not modeled. We did not address projected land-use changes.

### Avian Distribution Models

Breeding-season point-count survey data were used to build distribution models for 60 avian focal species (Table S1). Species presence and absence data were aggregated at the 800-m pixel level for modeling purposes. For each species, we generated predictions of current and future distribution based on the vegetation and climate datasets described above, as well as stream proximity as a proxy for riparian vegetation. We used two distribution-modeling algorithms: maximum entropy (Maxent 3.2.1) [59] and generalized additive models (GAM) [60]. Although Maxent is typically used with presence-only data, we incorporated species absence data in place of random environmental background data to constrain the models to the environmental space that was sampled [61]. We used default program settings except for the regularization value, which was increased from 1 to 2 to reduce over-fitting. We implemented generalized additive models using the ‘gam’ package for R with a binomial distribution and logit link function, using smoothing splines with no target degrees of freedom specified.

Receiver operating characteristic (ROC) plots [62] based on presence and absence data were constructed for each model. The ROC area under the curve (AUC) values for randomly selected test locations (25% of data withheld from models) were compared to evaluate model performance across classification thresholds. Model predictions for the current period were also visually inspected and compared to expert-based range maps.

### Modern Analog Analysis

Using version 0.6–6 of the ‘analogue’ package [63] for R, we conducted an analysis of modern analogs [35] for resulting model predictions. Due to computational limitations at the 800-m resolution of our predictions, we averaged predicted probabilities of species occurrence across 10-km grid cells and conducted the modern analog analysis on the resulting values.

We calculated a Bray-Curtis distance (dissimilarity) metric for each pair of current grid cells (n = 6,375), based on predicted probability of occurrence for each species. To determine an appropriate threshold for determining “analog” vs. “non-analog” communities, we employed an objective approach that involved identifying the value of the dissimilarity metric (*d*) that best separated current avian assemblages from each other [64]. Because we had no *a priori* classification of avian communities for California, except at the broad-scale level of general habitat types (e.g., conifer, oak woodland), we used the Bray-Curtis dissimilarity matrix to group current grid cells using a hierarchical agglomerative cluster analysis based on Ward's criterion [65]. As a starting point, we clustered current grid cells into 60 groups, which is similar to the number of wildlife habitat types defined by the State of California [56], and also coincides with the number of focal species modeled. To bracket this definition of an avian community, we also evaluated clusters with 20 and 100 groups (Figs. S3 and S4). The lower value represents a broad scale of species assembly and, when mapped, is at the spatial scale of a general vegetation association or ecological subregion. The upper value is a relatively fine-scale representation of avian assemblages, and is most likely to represent the local community scale at which species interactions have the largest influence [44], [66].

Using the grid cell groupings identified by the cluster analysis, we used a receiver operating characteristic (ROC) curve analysis to identify the dissimilarity value for which the true positive rate within groups (or “analogs”) was maximized and false positive rate (between groups, or “non-analogs”) was minimized; this is equivalent to the point at which a line drawn tangent to the curve has a slope of one [64]. Between- and within-group dissimilarity values were combined across groups to develop a single ROC curve and identify a single optimal dissimilarity value. This analysis was limited to the *k* nearest neighbors (grid cells of lowest dissimilarity), where *k* was specified as the minimum group size. We chose the highest value of *k* that could separate within- and among-group dissimilarity values to ensure that our definition of non-analog was as conservative as possible. This optimal dissimilarity threshold value (*p*) was determined separately for each SDM algorithm (Maxent, GAM) and each grouping level (20–60–100). The value of *p* decreased as the number of groups increased (Table S4).

For each community grouping level, climate model, and distribution modeling algorithm, we calculated the number of modern analogs for each future grid cell based on the number of current grid cells with which Bray-Curtis dissimilarity was less than *p*. The percent of novel grid cells was summarized for each of the resulting grid layers. We calculated the percent of grid cells with effectively zero (<0.5%) modern analogs, thereby standardizing this calculation according to the total number of grid cells available (6,375 for the 10-km resolution). A comparison across a range of scales (10–50 km) suggested that these standardized results were relatively consistent (within 5% of each other) across scales, while the percent of grid cells with exactly zero modern analogs increased with grid-cell size.

### Species Turnover

For comparison with our quantification of no-analog communities, and to characterize the geographic patterns of changes in community composition (or species turnover) between the current and future periods, we also calculated the change in community composition over time. For each 10-km grid cell, we calculated the Bray-Curtis distance or dissimilarity metric between current and future community composition, based on predicted probability of occurrence for the same 60 avian focal species.

## Supporting Information

Figure S1 Locations and sources of point-count data used to develop avian distribution models. Occurrence information from 16,742 point-count locations was aggregated for each species at the 800-m pixel level for modelling purposes, resulting in an effective sample size of 6,964. PRBO  =  PRBO Conservation Science; RSL  =  USDA Forest Service Redwood Sciences Lab; KBO  =  Klamath Bird Observatory; BBS  =  North American Breeding Bird Survey.
(3.51 MB TIF)

Figure S2 Modeled current and future vegetation distribution for California. A, current vegetation. B, future vegetation based on GFDL CM2.1, Scenario A2, 2038–2070. C, future vegetation based on NCAR CCSM3.0, Scenario A2, 2038–2069. Models were developed using California Gap Analysis vegetation data (see Table S2 for definitions of vegetation codes). Versions used as inputs to bird models also included current urban, agricultural, and wetland/riparian vegetation types (from Gap Analysis data).
(4.69 MB TIF)

Figure S3 Levels of bird community aggregation used to determine optimal no-analog thresholds for generalized additive model predictions. A, 20 groups. B, 60 groups. C, 100 groups.
(3.84 MB TIF)

Figure S4 Levels of bird community aggregation used to determine optimal no-analog thresholds for maximum entropy model predictions. A, 20 groups. B, 60 groups. C, 100 groups.
(3.92 MB TIF)

Table S1 Focal species, habitat categories, and migratory status.
(0.10 MB DOC)

Table S2 Vegetation classes modeled.
(0.04 MB DOC)

Table S3 Summary of bioclimatic and soil variables included in vegetation classification models^1^.
(0.05 MB DOC)

Table S4 Optimal dissimilarity thresholds by level of community aggregation (number of groups) and distribution model algorithm.
(0.04 MB DOC)
