# Supplementary figures and images for: Re-Shuffling of Species with Climate Disruption: A No-Analog Future for California Birds?
Source: PLoS One. 2009 Sep 2;4(9):e6825. doi: 10.1371/journal.pone.0006825 (PMC2730567; doi:10.1371/journal.pone.0006825)

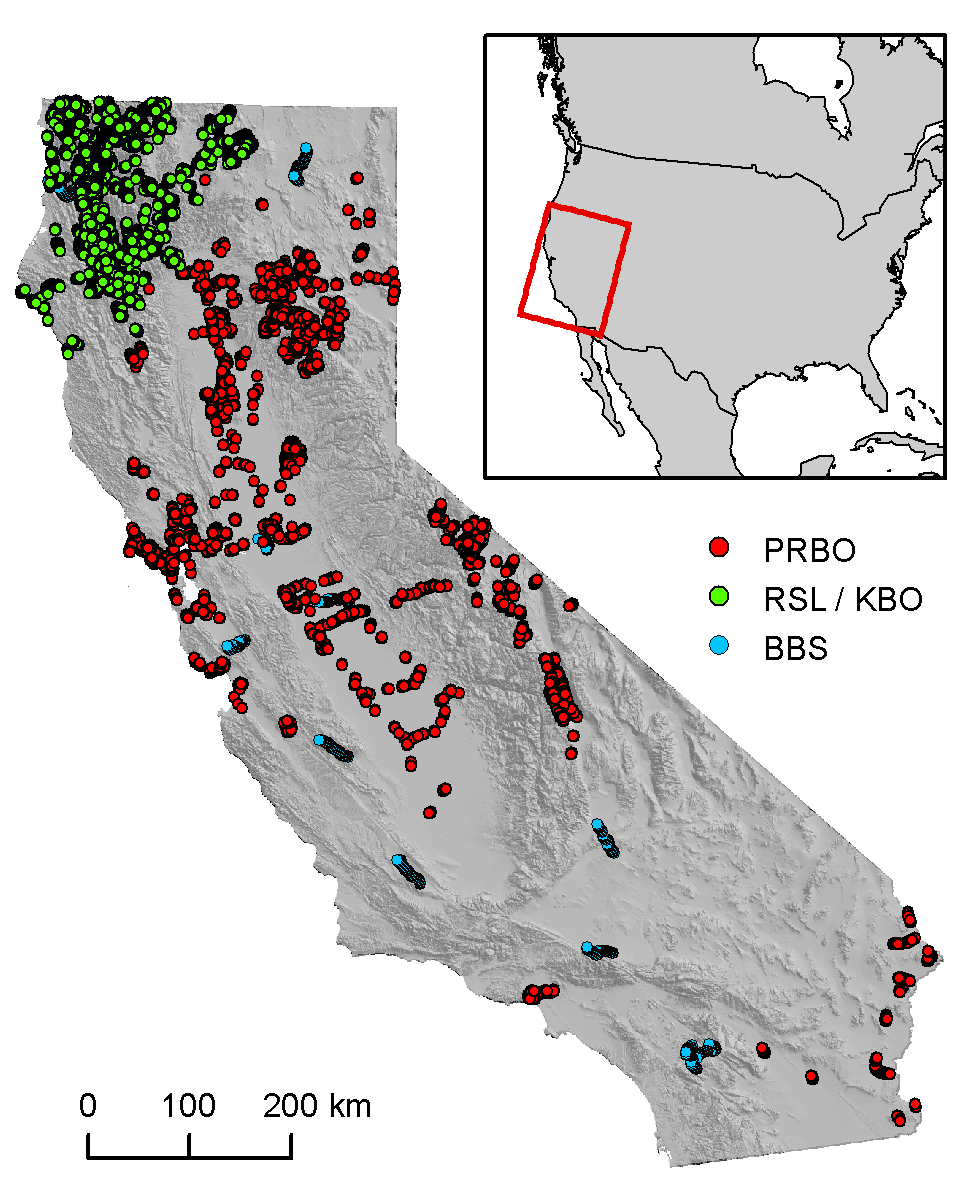

Supplement: Figure S1 — Locations and sources of point-count data used to develop avian distribution models. Occurrence information from 16,742 point-count locations was aggregated for each species at the 800-m pixel level for modelling purposes, resulting in an effective sample size of 6,964. PRBO = PRBO Conservation Science; RSL = USDA Forest Service Redwood Sciences Lab; KBO = Klamath Bird Observatory; BBS = North American Breeding Bird Survey. (3.51 MB TIF) [file pone.0006825.s001.tif]

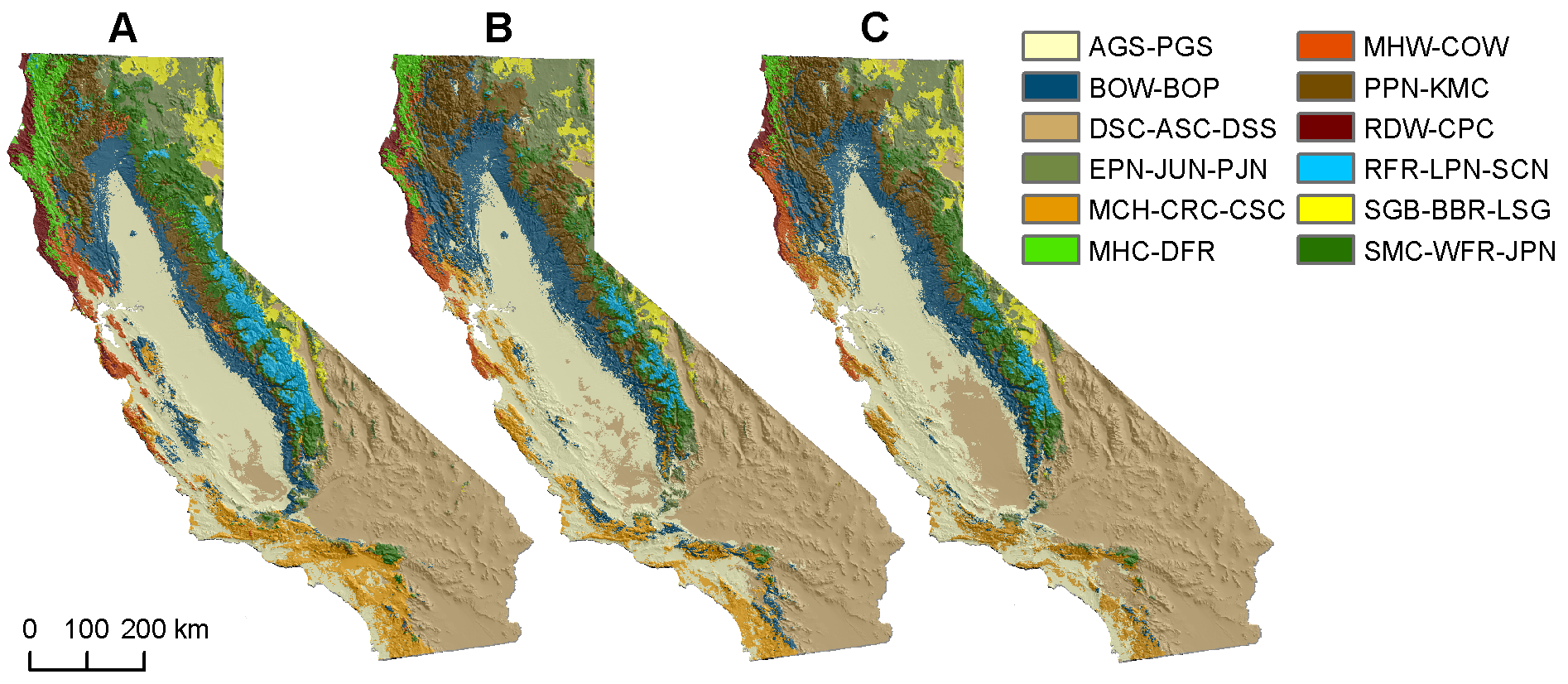

Supplement: Figure S2 — Modeled current and future vegetation distribution for California. A, current vegetation. B, future vegetation based on GFDL CM2.1, Scenario A2, 2038–2070. C, future vegetation based on NCAR CCSM3.0, Scenario A2, 2038–2069. Models were developed using California Gap Analysis vegetation data (see Table S2 for definitions of vegetation codes). Versions used as inputs to bird models also included current urban, agricultural, and wetland/riparian vegetation types (from Gap Analysis data). (4.69 MB TIF) [file pone.0006825.s002.tif]

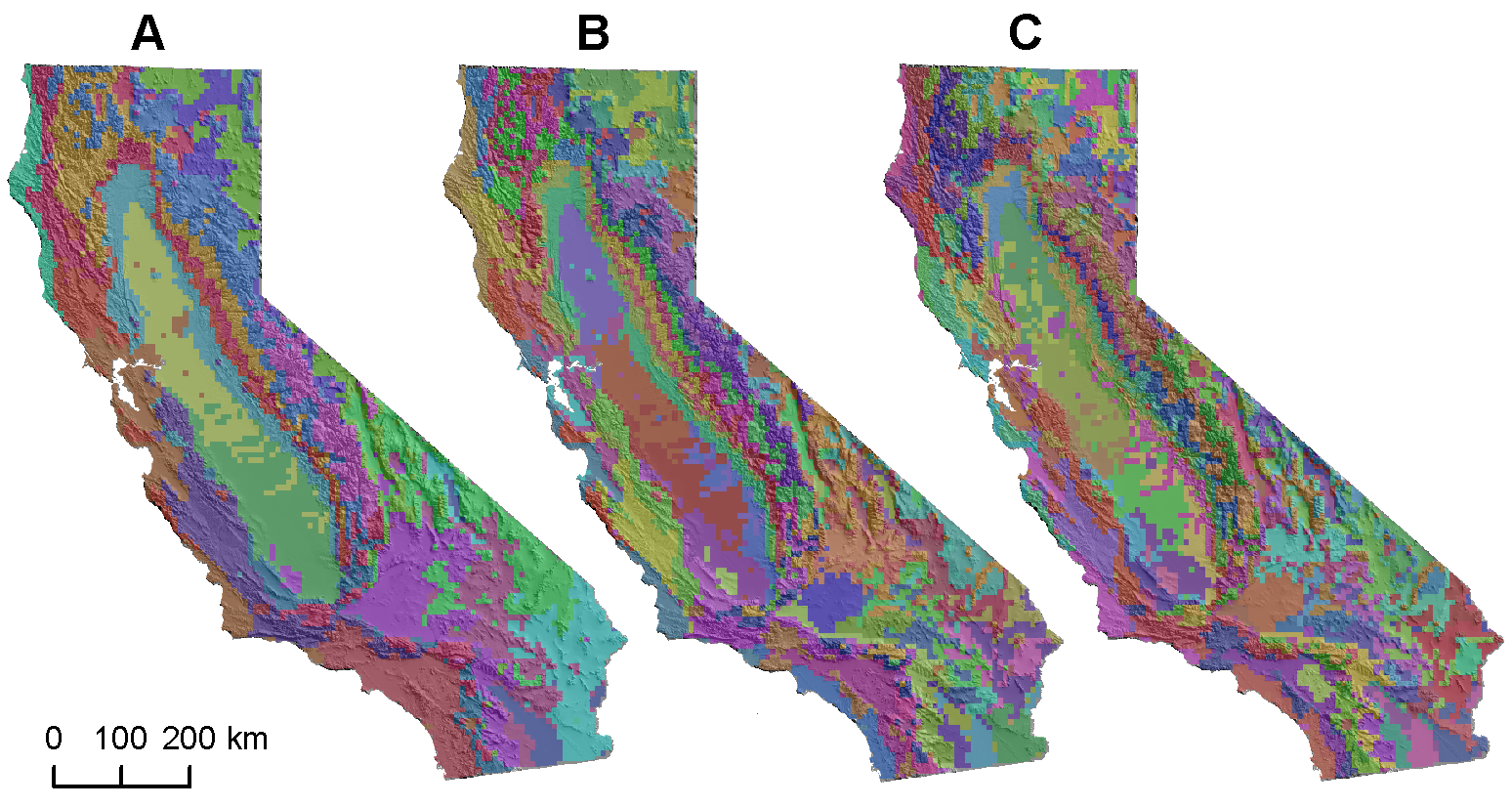

Supplement: Figure S3 — Levels of bird community aggregation used to determine optimal no-analog thresholds for generalized additive model predictions. A, 20 groups. B, 60 groups. C, 100 groups. (3.84 MB TIF) [file pone.0006825.s003.tif]

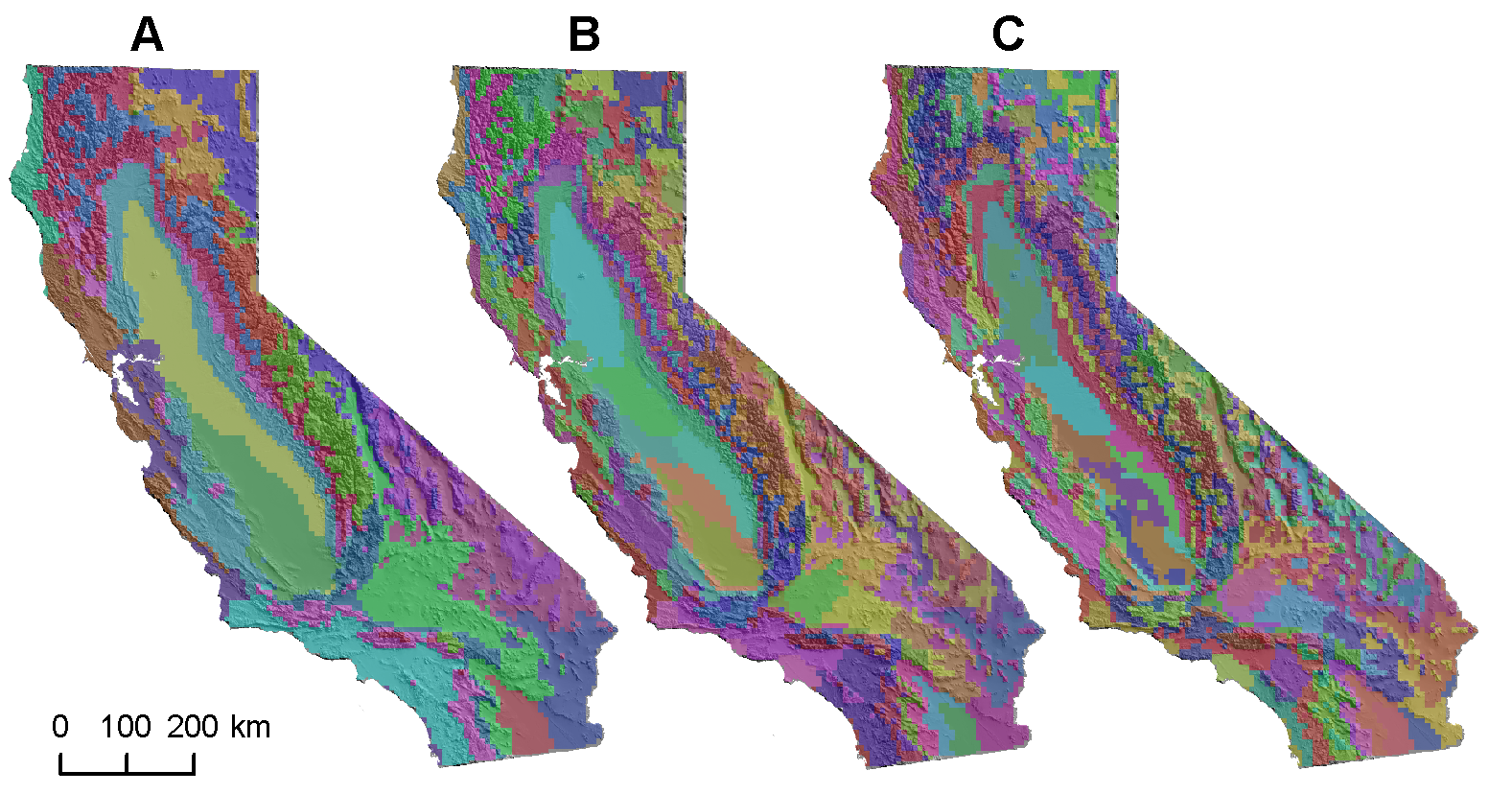

Supplement: Figure S4 — Levels of bird community aggregation used to determine optimal no-analog thresholds for maximum entropy model predictions. A, 20 groups. B, 60 groups. C, 100 groups. (3.92 MB TIF) [file pone.0006825.s004.tif]
