# Supplementary material for: Re-Shuffling of Species with Climate Disruption: A No-Analog Future for California Birds?
Source: PLoS One. 2009 Sep 2;4(9):e6825. doi: 10.1371/journal.pone.0006825 (PMC2730567; doi:10.1371/journal.pone.0006825)
Supplement: Table S1 — Focal species, habitat categories, and migratory status. (0.10 MB DOC) [file pone.0006825.s005.doc]

**Table S1. Focal species, habitat categories, and migratory status.**

| Common name | Scientific name | Habitat^1^ | Migratory status^2^ |
| --- | --- | --- | --- |
| Wood Duck | *Aix sponsa* | O | R |
| Mountain Quail | *Oreortyx pictus* | C, S | R |
| California Quail | *Callipepla californica* | O, S | R |
| Band-tailed Pigeon | *Columba fasciata* | O | R |
| Turkey Vulture | *Cathartes aura* | S | R |
| White-tailed Kite | *Elanus leucurus* | G | R |
| Red-shouldered Hawk | *Buteo lineatus* | O | R |
| Greater Roadrunner | *Geococcyx californianus* | S | R |
| Nuttall's Woodpecker | *Picoides nuttallii* | O | R |
| Black-backed Woodpecker | *Picoides arcticus* | C | R |
| Pileated Woodpecker | *Dryocopus pileatus* | C | R |
| Acorn Woodpecker | *Melanerpes formicivorus* | O | R |
| Lewis's Woodpecker | *Melanerpes lewis* | O | R |
| Vaux's Swift | *Chaetura vauxi* | C | M, D |
| Costa's Hummingbird | *Calypte costae* | S | M |
| Allen's Hummingbird | *Selasphorus sasin* | S | M |
| Ash-throated Flycatcher | *Myiarchus cinerascens* | O | M |
| Gray Flycatcher | *Empidonax wrightii* | C | M, D |
| Yellow-billed Magpie | *Pica nuttalli* | O | R |
| Steller's Jay | *Cyanocitta stelleri* | C | R |
| Western Scrub-Jay | *Aphelocoma californica* | O, S | R |
| Gray Jay | *Perisoreus canadensis* | C | R |
| Clark's Nutcracker | *Nucifraga columbiana* | C | R |
| Western Meadowlark | *Sturnella neglecta* | G | R |
| Purple Finch | *Carpodacus purpureus* | C | R |
| Cassin's Finch | *Carpodacus cassinii* | C | R |
| Savannah Sparrow | *Passerculus sandwichensiss* | G | M |
| Lark Sparrow | *Chondestes grammacus* | O | R |
| White-crowned Sparrow | *Zonotrichia leucophrys* | S | M, D |
| Chipping Sparrow | *Spizella passerina* | C | M, D |
| Oregon Junco | *Junco hyemalis oreganus* | C | R |
| Sage Sparrow | *Amphispiza belli* | S | R |
| Rufous-crowned Sparrow | *Aimophila ruficeps* | S | R |
| Song Sparrow | *Melospiza melodia* | R, S | R |
| Fox Sparrow | *Passerella iliaca* | C | M |
| Spotted Towhee | *Pipilo maculatus* | S | R |
| California Towhee | *Pipilo crissalis* | O, S | R |
| Black-headed Grosbeak | *Pheuticus melanocephalus* | R | M, D |
| Blue Grosbeak | *Guiraca caerulea* | R | M |
| Western Tanager | *Piranga ludoviciana* | C | M, D |
| Tree Swallow | *Tachycineta bicolor* | R | M, D |
| Warbling Vireo | *Vireo gilvus* | R | M, D |
| Hutton's Vireo | *Vireo huttoni* | O | R |
| Yellow-rumped Warbler | *Dendroica coronata auduboni* | C | M, D |
| Black-throated Gray Warbler | *Dendroica nigrescens* | C | M, D |
| MacGillivray's Warbler | *Oporornis tolmiei* | C | M, D |
| Common Yellowthroat | *Geothlypis trichas* | R, S | R |
| Wilson's Warbler | *Wilsonia pusilla* | R | M, D |
| California Thrasher | *Toxostoma redivivum* | O, S | R |
| Cactus Wren | *Campylorhynchus brunneicapillus* | S | R |
| Bewick's Wren | *Thryomanes bewickii* | O, S | R |
| Brown Creeper | *Certhia americana* | C | R |
| White-breasted Nuthatch | *Sitta carolinensis* | O | R |
| Red-breasted Nuthatch | *Sitta canadensis* | C | R |
| Oak Titmouse | *Baeolophus inornatus* | O | R |
| Wrentit | *Chamaea fasciata* | S | R |
| Golden-crowned Kinglet | *Regulus satrapa* | C | R |
| Blue-gray Gnatcatcher | *Polioptila caerulea* | O, S | M, D |
| Varied Thrush | *Ixoreus naevius* | C | M |
| Western Bluebird | *Sialia mexicanus* | O | R |

^1^ O = oak woodland, C = coniferous forest, S = scrub, G = grassland, R = riparian. R = year-round resident

^2^ M = long- or short-distance migrant, D = desert-breeding migrant.
