# Supplementary material for: Re-Shuffling of Species with Climate Disruption: A No-Analog Future for California Birds?
Source: PLoS One. 2009 Sep 2;4(9):e6825. doi: 10.1371/journal.pone.0006825 (PMC2730567; doi:10.1371/journal.pone.0006825)
Supplement: Table S2 — Vegetation classes modeled. (0.04 MB DOC) [file pone.0006825.s006.doc]

Table S2. Vegetation classes modeled.

|  | California Wildlife Habitat Relationships vegetation types (codes) |
| --- | --- |
| 1 | Annual Grassland (AGS), Perennial Grassland (PGS) |
| 2 | Blue Oak Woodland (BOW), Blue Oak-Foothill Pine (BOP) |
| 3 | Desert Scrub (DSC), Alkali Desert Scrub (ASC), Desert Succulent Shrub (DSS) |
| 4 | Eastside Pine (EPN), Juniper (JUN), Pinyon-Juniper (PJN) |
| 5 | Mixed Chaparral (MCH), Chamise-Redshank Chaparral (CRC), Coastal Scrub (CSC) |
| 6 | Montane Hardwood-Conifer (MHC), Douglas Fir (DFR) |
| 7 | Montane Hardwood (MHW), Coastal Oak Woodland (COW) |
| 8 | Ponderosa Pine (PPN), Klamath Mixed Conifer (KMC) |
| 9 | Redwood (RWD), Closed-Cone Pine Cypress (CPC) |
| 10 | Red Fir (RFR), Lodgepole Pine (LPN), Subalpine Conifer (SCN) |
| 11 | Sagebrush (SGB), Bitterbrush (BBR), Low Sage (LSG) |
| 12 | Sierran Mixed Conifer (SMC), White Fir (WFR), Jeffrey Pine (JPN) |
