# Supplementary material for: Re-Shuffling of Species with Climate Disruption: A No-Analog Future for California Birds?
Source: PLoS One. 2009 Sep 2;4(9):e6825. doi: 10.1371/journal.pone.0006825 (PMC2730567; doi:10.1371/journal.pone.0006825)
Supplement: Table S3 — Summary of bioclimatic and soil variables included in vegetation classification models1. (0.05 MB DOC) [file pone.0006825.s007.doc]

| Variable name | Definition | Current, 1970-1999 | NCAR CCSM3.0, Scenario A2, 2038-2069 | GFDL CM2.1, Scenario A2, 2038-2070 |
| --- | --- | --- | --- | --- |
| Bio_1 | Annual mean temperature (˚C) | 14.2 ± 4.6 | 15.9 ± 4.6 | 16.4 ± 4.6 |
| Bio_2 | Mean diurnal range (˚C) (mean of monthly temperature range) | 14.6 ± 2.1 | 14.8 ± 2.1 | 14.7 ± 2.1 |
| Bio_3 | Isothermality ((Bio_2/Bio_7^2^) * 100) | 45.1 ± 5.5 | 42.7 ± 5.4 | 42.5 ± 5.3 |
| Bio_4 | Temperature seasonality (standard deviation *100) | 630.9 ± 124.2 | 682.6 ± 134.6 | 700.7 ± 133.4 |
| Bio_10 | Mean temperature of warmest quarter (˚C) | 22.5 ± 5.1 | 25.0 ± 5.0 | 25.6 ± 5.1 |
| Bio_12 | Annual precipitation (mm * 10) | 602.9 ± 541.2 | 418.5 ± 378.2 | 562.4 ± 496.0 |
| Bio_15 | Precipitation seasonality (coefficient of variation) | 77.5 ± 16.4 | 84.2 ± 14.6 | 90.4 ± 17.7 |
| Bio_17 | Precipitation of driest quarter (mm * 10) | 20.2 ± 19.5 | 11.6 ± 12.1 | 14.3 ± 15.8 |
| Sol_rad | Average yearly solar radiation (watt-hours / m^2^) | 165,611 ± 70,311 | - | - |
| Slope | Maximum rate of change in elevation (degrees) | 22.9 ± 20.3 | - | - |
| Soil_pH | Soil acidity/alkalinity (pH) | 6.6 ± 1.1 | - | - |
| Soil_perm | Soil permeability (inches of H_2_0 held / inch of soil) | 9.2 ± 8.4 | - | - |
| Soil_wat | Soil available water capacity (inches of H_2_0 / hour) | 8.7 ± 9.5 | - | - |

^1^ Mean (± SD) values were summarized for the state of California.

^2^ Temperature annual range (maximum temperature of warmest month - minimum temperature of coldest month).
