# Supplementary material for: Re-Shuffling of Species with Climate Disruption: A No-Analog Future for California Birds?
Source: PLoS One. 2009 Sep 2;4(9):e6825. doi: 10.1371/journal.pone.0006825 (PMC2730567; doi:10.1371/journal.pone.0006825)
Supplement: Table S4 — Optimal dissimilarity thresholds by level of community aggregation (number of groups) and distribution model algorithm. (0.04 MB DOC) [file pone.0006825.s008.doc]

|  | Generalized Additive Model |  | Maximum Entropy Model |  |
| --- | --- | --- | --- | --- |
|  | *p*^1^ | *k*^2^ | *p*^1^ | *k*^2^ |
| 5 groups | 0.403 | 337 | 0.421 | 743 |
| 20 groups | 0.319 | 89 | 0.256 | 94 |
| 60 groups | 0.285 | 37 | 0.203 | 22 |
| 100 groups | 0.240 | 14 | 0.192 | 14 |

^1^ The dissimilarity value for which the true positive rate within current groups was maximized and false positive rate was minimized.

^2^ The number of nearest neighbors (*k*) within and among groups used to identify *p* (equivalent to the minimum group size).
